# Association of GLIM-defined malnutrition with postoperative complications in older patients undergoing hip arthroplasty

**DOI:** 10.1097/MD.0000000000048892

**Published:** 2026-05-15

**Authors:** Zhixing Lu, Wenwei Xie

**Affiliations:** a Department of Joint and Trauma Surgery, The First Dongguan Affiliated Hospital of Guangdong Medical University, Tangxia, Dongguan, Guangdong Province, P.R.China; b Department of Orthopedics, Dongguan Songshanhu Central Hospital Affiliated to Guangdong Medical University, Dongguan City, Guangdong Province, P.R.China.

**Keywords:** global leadership initiative on malnutrition, hip arthroplasty, malnutrition, nutritional assessment, older adults, postoperative complications

## Abstract

Malnutrition is common in older patients undergoing hip arthroplasty and may adversely affect postoperative recovery. The global leadership initiative on malnutrition (GLIM) criteria provide a standardized framework for nutritional diagnosis, but evidence regarding their prognostic value in older hip arthroplasty patients remains limited. This study evaluated the association between GLIM-defined malnutrition and postoperative complications in this population. This retrospective observational study included patients aged 60 years or older who underwent primary hip arthroplasty between January 2022 and December 2025. Nutritional status was assessed using the GLIM criteria. Patients with malnutrition were matched 1:2 with those without malnutrition according to age, sex, surgical year, and primary indication for hip arthroplasty. The primary outcome was postoperative complications during hospitalization or within 30 days after surgery. Secondary outcomes included length of hospital stay, intensive care unit admission, and perioperative blood transfusion. Univariable and multivariable logistic regression analyses were performed to identify factors associated with postoperative complications. The matched cohort comprised 168 patients, including 56 with malnutrition and 112 without malnutrition. The malnutrition group had lower body weight, body mass index, hemoglobin, albumin, total lymphocyte count, and total protein, as well as higher C-reactive protein levels. Postoperative complications occurred more frequently in the malnutrition group than in the non-malnutrition group (42.9% vs 20.5%, *P* = .002). Length of hospital stay and perioperative blood transfusion rate were also higher in the malnutrition group. Multivariable analysis showed that GLIM-defined malnutrition was independently associated with postoperative complications (adjusted odds ratio = 2.74, 95% confidence interval: 1.23–6.09, *P* = .013). GLIM-defined malnutrition was independently associated with postoperative complications in older patients undergoing hip arthroplasty. Preoperative GLIM-based nutritional assessment may improve perioperative risk stratification in this population.

## 1. Introduction

Hip arthroplasty, including total hip arthroplasty (THA) and hemiarthroplasty (HA), is a standard surgical procedure for older adults with femoral neck fracture, osteonecrosis of the femoral head, advanced osteoarthritis, and other disabling hip disorders. In this population, perioperative management is complicated by advanced age, multimorbidity, impaired physiologic reserve, and a high prevalence of nutritional disorders, all of which may affect postoperative recovery, complication burden, and healthcare utilization. Recent evidence has shown that nutritional disorders are common across hip and knee arthroplasty populations, with particularly substantial prevalence in hip fracture arthroplasty cohorts, while heterogeneity in diagnostic definitions remains a major barrier to consistent risk stratification.^[1-3]^ Malnutrition in older orthopedic patients is clinically relevant because it is closely linked to impaired immune function, reduced muscle mass and strength, delayed wound healing, diminished rehabilitation capacity, and increased vulnerability to postoperative medical complications. Contemporary studies in arthroplasty and related geriatric orthopedic settings have shown that nutritional impairment is associated with adverse outcomes such as surgical site infection, periprosthetic joint infection, prolonged hospitalization, delirium, and impaired functional recovery. A recent meta-analysis further reported that malnutrition significantly increased the risk of surgical site infection and periprosthetic joint infection after total joint arthroplasty.^[4-6]^

Despite growing recognition of perioperative nutrition as a modifiable determinant of outcome, nutritional assessment in routine orthopedic practice is still often based on isolated surrogate markers such as serum albumin, lymphocyte count, or transferrin. Recent studies have confirmed that abnormalities in these indices remain frequent in older arthroplasty populations and are associated with nutritional vulnerability, but they do not fully capture the multidimensional nature of malnutrition. In parallel, increasing attention has been directed toward related constructs such as sarcopenia and geriatric nutritional risk, both of which have been associated with transfusion requirement, functional decline, and less favorable postoperative outcomes after hip or knee arthroplasty.^[7,8]^ The global leadership initiative on malnutrition (GLIM) framework provides a more standardized diagnostic approach by integrating phenotypic criteria, including weight loss, low body mass index (BMI), and reduced muscle mass, with etiologic criteria such as reduced intake, impaired assimilation, and inflammation or disease burden. This structure may be particularly suitable for older patients undergoing hip arthroplasty, in whom malnutrition frequently coexists with frailty, sarcopenia, anemia, and inflammatory activation. However, although perioperative nutritional optimization strategies, including oral nutritional supplementation, are increasingly being explored in geriatric hip surgery, evidence specifically evaluating GLIM-defined malnutrition in older hip arthroplasty cohorts remains limited.^[9,10]^

Accordingly, the present study investigated the clinical significance of GLIM-defined malnutrition in older patients undergoing hip arthroplasty and examined its relationship with postoperative complications and short-term in-hospital outcomes. By applying a structured nutritional diagnostic framework within a matched cohort, this study sought to provide further evidence for perioperative risk assessment and nutritional screening in geriatric hip arthroplasty practice.

## 2. Materials and methods

### 2.1. Study design

This study was approved by the Ethics Committee of Dongguan Songshanhu Central Hospital Affiliated to Guangdong Medical University. This retrospective observational study included older patients who underwent hip arthroplasty at our institution between January 2022 and December 2025. Eligible participants were patients aged 60 years or older who received primary hip arthroplasty, including THA or HA, for hip-related disorders and had complete preoperative clinical, nutritional, and perioperative records available for analysis. Nutritional status was assessed according to the GLIM criteria, and the association between GLIM-defined malnutrition and postoperative complications was subsequently evaluated. Patients were excluded if they had undergone revision hip arthroplasty, had multiple fractures or polytrauma, had concurrent malignant disease, severe hematologic disorders, active infection, or end-stage hepatic or renal failure, or if key medical records were incomplete or missing. Patients with severe cognitive impairment or other conditions preventing reliable nutritional assessment were also excluded. This study was conducted in accordance with the principles of the Declaration of Helsinki and was approved by the Institutional Medical Ethics Committee of our hospital. Written informed consent was obtained from all patients or their authorized representatives prior to treatment.

### 2.2. Data collection

Demographic, clinical, perioperative, and laboratory data were extracted retrospectively from the electronic medical record system of our institution by trained investigators using a standardized data collection form. Baseline demographic variables included age, sex, body weight, height, and BMI. Information on lifestyle and social characteristics, including smoking history, alcohol consumption history, marital status, and living status, was also collected when available. Comorbidity-related data included the presence of hypertension, diabetes mellitus, coronary artery disease, chronic obstructive pulmonary disease, chronic kidney disease, cerebrovascular disease, and other major coexisting medical conditions. In addition, preoperative physical status was evaluated according to the American Society of Anesthesiologists (ASA) classification. Perioperative variables were also recorded, including surgical procedure type, categorized as THA or HA, primary surgical indication such as femoral neck fracture, femoral head necrosis, osteoarthritis, or other hip disorders, and anesthesia modality. Operative duration, intraoperative blood loss, perioperative blood transfusion, and length of hospital stay were collected as perioperative parameters. Laboratory indices obtained from preoperative routine blood and biochemical examinations included hemoglobin, serum albumin, total lymphocyte count, total protein, serum creatinine, and C-reactive protein. All laboratory measurements were derived from the most recent preoperative test results available before surgery.

### 2.3. Nutritional assessment

Nutritional status was assessed according to the GLIM criteria. In accordance with the GLIM framework, malnutrition was diagnosed when at least one phenotypic criterion and 1 etiologic criterion were present. The phenotypic criteria included non-volitional weight loss, low BMI, and reduced muscle mass, whereas the etiologic criteria included reduced food intake or impaired assimilation and the presence of inflammation or disease burden. Specifically, non-volitional weight loss was defined as >5% within the previous 6 months or >10% beyond 6 months. Low BMI was defined as <20.0 kg/m^2^ in patients aged <70 years and <22.0 kg/m^2^ in those aged ≥70 years. Reduced food intake or impaired assimilation was identified from documented decreased dietary intake, prolonged insufficient intake, or gastrointestinal conditions affecting nutrient absorption. Inflammation or disease burden was determined according to the presence of acute or chronic disease conditions associated with inflammatory activity. Based on the GLIM assessment, patients were classified into the malnutrition group, and patients without malnutrition were subsequently matched at a 1:2 ratio according to age, sex, surgical year, and the primary indication for hip arthroplasty for further analyses.

### 2.4. Outcome definitions

The primary outcome of this study was the occurrence of postoperative complications during hospitalization or within 30 days after surgery. Postoperative complications were defined as any clinically documented adverse event occurring after hip arthroplasty, including surgical site infection, pneumonia, urinary tract infection, lower-extremity deep vein thrombosis, pulmonary embolism, delayed wound healing, cardiovascular events, delirium, pressure injury, readmission, or reoperation. Surgical site infection was diagnosed according to clinical manifestations, laboratory findings, and microbiological evidence when available. Pneumonia and urinary tract infection were identified on the basis of clinical symptoms, imaging findings, and relevant laboratory or microbiological results. Deep vein thrombosis and pulmonary embolism were confirmed by imaging examinations. Delayed wound healing referred to persistent wound exudation, dehiscence, or failure of the incision to heal within the expected postoperative period. Cardiovascular events included newly developed serious cardiac or cerebrovascular complications requiring medical intervention. Delirium was diagnosed according to postoperative clinical assessment documented in the medical records. Pressure injury was defined as localized skin and/or soft tissue damage occurring during the perioperative hospitalization period.

Secondary outcomes included prolonged length of hospital stay, intensive care unit (ICU) admission, blood transfusion rate, and total hospitalization cost. Length of hospital stay was calculated as the number of days from admission to discharge. ICU admission referred to any postoperative transfer to the ICU for further monitoring or treatment. Blood transfusion rate was defined as the proportion of patients who received allogeneic blood transfusion during the perioperative period. Total hospitalization cost was calculated on the basis of the final inpatient billing record.

### 2.5. Statistical analysis

All statistical analyses were performed using IBM SPSS Statistics, version 30.0 (IBM Corp., Armonk). Continuous variables were expressed as mean ± standard deviation and compared using the independent-samples t test. Categorical variables were presented as number (%) and compared using the chi-square test or Fisher’s exact test, as appropriate. Patients without malnutrition were matched to those with malnutrition at a 1:2 ratio according to age, sex, surgical year, and the primary indication for hip arthroplasty. For each malnourished patient, 2 non-malnourished controls were selected with identical sex, surgical year category (2022, 2023, 2024, or 2025), and primary indication for hip arthroplasty, and the closest age within a caliper of ±2 years.

Univariable logistic regression analysis was performed to identify factors associated with postoperative complications. Variables with statistical significance in the univariable analysis, together with clinically relevant factors, were subsequently entered into a multivariable logistic regression model to determine independent factors associated with postoperative complications. The results of logistic regression were reported as odds ratios with 95% confidence intervals. All tests were 2-sided, and a *P* value < .05 was considered statistically significant.

## 3. Results

### 3.1. GLIM classification and matching results

According to the GLIM criteria, 56 patients were classified into the malnutrition group. To reduce potential confounding, patients in the non-malnutrition group were matched to those in the malnutrition group at a 1:2 ratio based on age, sex, year of surgery, and the primary indication for hip arthroplasty. Following matching, 112 patients without malnutrition were included in the final analysis. Therefore, the matched study cohort comprised a total of 168 patients, including 56 patients in the malnutrition group and 112 patients in the non-malnutrition group.

### 3.2. Comparison of baseline characteristics between the 2 matched groups

There were no significant differences in age (78.4 ± 7.2 vs 77.9 ± 7.0 years, *P* = .669), sex distribution (35.7% vs 38.4% male, *P* = .866), surgical year (*P* = .999), or primary indication for hip arthroplasty (*P* = .989). Among the remaining baseline characteristics, patients in the malnutrition group had lower body weight (54.8 ± 8.9 vs 59.6 ± 9.4 kg, *P* = .002) and lower BMI (21.1 ± 2.8 vs 22.4 ± 2.9 kg/m^2^, *P* = .006) than those in the non-malnutrition group. The proportion of patients living alone was also higher in the malnutrition group (33.9% vs 18.8%, *P* = .047). No significant between-group differences were observed in height, smoking history, alcohol consumption history, marital status, comorbidities, or ASA class (all *P* > .05; Table 1).

**Table 1:** Comparison of baseline characteristics between the malnutrition group and the matched non-malnutrition group.

Variable	Malnutrition group (n = 56)	Non-malnutrition group (n = 112)	Test statistic	*P* value
General demographic characteristics				
Age, yr	78.4 ± 7.2	77.9 ± 7.0	*t* = 0.43	.669
Male sex, n (%)	20 (35.7)	43 (38.4)	χ^2^ = 0.03	.866
Body weight, kg	54.8 ± 8.9	59.6 ± 9.4	*t* = −3.23	.002
Height, cm	161.2 ± 8.1	163.0 ± 7.6	*t* = −1.39	.169
BMI, kg/m^2^	21.1 ± 2.8	22.4 ± 2.9	*t* = −2.80	.006
Smoking history, n (%)	17 (30.4)	31 (27.7)	χ^2^ = 0.03	.856
Alcohol consumption history, n (%)	11 (19.6)	24 (21.4)	χ^2^ = 0.00	.946
Marital status, n (%)				
Married	38 (67.9)	88 (78.6)		
Widowed	14 (25.0)	17 (15.2)	χ^2^ = 2.57	.277
Other	4 (7.1)	7 (6.2)		
Living alone, n (%)	19 (33.9)	21 (18.8)	χ^2^ = 3.94	.047
Matching-related variables				
Surgical year, n (%)				
2022	9 (16.1)	18 (16.1)		
2023	14 (25.0)	29 (25.9)		
2024	17 (30.4)	34 (30.4)	χ^2^ = 0.02	.999
2025	16 (28.6)	31 (27.7)		
Primary indication for hip arthroplasty, n (%)				
Femoral neck fracture	28 (50.0)	56 (50.0)		
Femoral head necrosis	15 (26.8)	32 (28.6)		
Osteoarthritis	9 (16.1)	16 (14.3)	χ^2^ = 0.12	.989
Other hip disorders	4 (7.1)	8 (7.1)		
Comorbidities and preoperative status				
Hypertension, n (%)	31 (55.4)	53 (47.3)	χ^2^ = 0.67	.413
Diabetes mellitus, n (%)	16 (28.6)	24 (21.4)	χ^2^ = 0.69	.405
Coronary artery disease, n (%)	14 (25.0)	19 (17.0)	χ^2^ = 1.06	.303
Chronic obstructive pulmonary disease, n (%)	9 (16.1)	10 (8.9)	χ^2^ = 1.25	.263
Chronic kidney disease, n (%)	8 (14.3)	10 (8.9)	χ^2^ = 0.63	.427
Cerebrovascular disease, n (%)	13 (23.2)	18 (16.1)	χ^2^ = 0.84	.361
Other major comorbidities, n (%)	12 (21.4)	17 (15.2)	χ^2^ = 0.63	.427
ASA class III–IV, n (%)	34 (60.7)	49 (43.8)	χ^2^ = 3.65	.056

ASA = American Society of Anesthesiologists, BMI = body mass index.

### 3.3. Comparison of perioperative variables and preoperative laboratory findings between the 2 matched groups

The 2 groups remained comparable with respect to surgical procedure, primary indication for hip arthroplasty, anesthesia modality, operative time, and intraoperative blood loss (all *P* > .05). However, patients in the malnutrition group had a significantly higher perioperative blood transfusion rate than those in the non-malnutrition group (32.1% vs 17.9%, *P* = .037), and their length of hospital stay was also longer (12.8 ± 4.7 vs 10.9 ± 3.9 days, *P* = .011). With regard to preoperative laboratory findings, the malnutrition group had significantly lower hemoglobin, albumin, total lymphocyte count, and total protein levels than the non-malnutrition group (all *P* ≤ .005). In contrast, serum creatinine did not differ significantly between groups (*P* = .257). C-reactive protein (CRP) was significantly higher in the malnutrition group than in the non-malnutrition group (12.9 ± 8.7 vs 9.8 ± 7.1 mg/L, *P* = .023; Table 2).

**Table 2:** Comparison of perioperative variables and preoperative laboratory findings between the malnutrition group and the matched non-malnutrition group.

Variable	Malnutrition group (n = 56)	Non-malnutrition group (n = 112)	Test statistic	*P* value
Surgical procedure, n (%)				
THA	22 (39.3)	49 (43.8)		
HA	34 (60.7)	63 (56.2)	χ^2^ = 0.30	.581
Primary indication for hip arthroplasty, n (%)				
Femoral neck fracture	28 (50.0)	56 (50.0)		
Femoral head necrosis	15 (26.8)	32 (28.6)		
Osteoarthritis	9 (16.1)	16 (14.3)		
Other hip disorders	4 (7.1)	8 (7.1)	χ^2^ = 0.12	.989
Anesthesia modality, n (%)				
General anesthesia	41 (73.2)	89 (79.5)		
Spinal anesthesia	13 (23.2)	20 (17.9)		
Combined spinal-epidural anesthesia	2 (3.6)	3 (2.7)	χ^2^ = 0.83	.659
Operative time, min	83.6 ± 18.4	80.9 ± 17.6	*t* = 0.91	.365
Intraoperative blood loss, mL	318.0 ± 108.0	286.0 ± 96.0	*t* = 1.88	.063
Perioperative blood transfusion, n (%)	18 (32.1)	20 (17.9)	χ^2^ = 4.35	.037
Length of hospital stay, d	12.8 ± 4.7	10.9 ± 3.9	*t* = 2.61	.011
Preoperative laboratory findings				
Hemoglobin, g/L	111.6 ± 15.8	118.9 ± 14.6	*t* = −2.89	.005
Albumin, g/L	34.7 ± 4.2	38.1 ± 3.8	*t* = −5.10	<.001
Total lymphocyte count, ×10^9^/L	1.42 ± 0.46	1.68 ± 0.51	*t* = −3.33	.001
Total protein, g/L	61.8 ± 5.9	65.0 ± 5.4	*t* = −3.41	.001
Serum creatinine, μmol/L	89.6 ± 24.8	85.1 ± 22.7	*t* = 1.14	.257
C-reactive protein, mg/L	12.9 ± 8.7	9.8 ± 7.1	*t* = 2.31	.023

CRP = C-reactive protein, HA = hemiarthroplasty, THA = total hip arthroplasty.

### 3.4. Comparison of postoperative complications between the 2 groups

In the matched cohort, postoperative complications occurred in 47 of 168 patients, corresponding to an overall incidence of 28.0%. The rate of any postoperative complication was significantly higher in the malnutrition group than in the non-malnutrition group (42.9% vs 20.5%, χ^2^ = 9.23, *P* = .002). Regarding individual postoperative complications, delirium was the most frequent event in both groups and occurred more often in the malnutrition group than in the non-malnutrition group (16.1% vs 6.3%), with borderline statistical significance (*P* = .052). Pneumonia and delayed wound healing were also numerically more common among patients with malnutrition (both 10.7% vs 3.6%, *P* = .085). In addition, the incidences of surgical site infection, lower-extremity deep vein thrombosis, readmission, and reoperation were higher in the malnutrition group, although these differences were not statistically significant. No significant between-group differences were observed for urinary tract infection or cardiovascular events (all *P* > .05; Table 3).

**Table 3:** Comparison of postoperative complications between the malnutrition group and the matched non-malnutrition group.

Variable	Malnutrition group (n = 56)	Non-malnutrition group (n = 112)	Test statistic	*P* value
Any postoperative complication, n (%)	24 (42.9)	23 (20.5)	χ^2^ = 9.23	.002
Surgical site infection, n (%)	4 (7.1)	3 (2.7)	Fisher’s exact test	.223
Pneumonia, n (%)	6 (10.7)	4 (3.6)	Fisher’s exact test	.085
Urinary tract infection, n (%)	5 (8.9)	5 (4.5)	Fisher’s exact test	.304
Lower-extremity deep vein thrombosis, n (%)	4 (7.1)	3 (2.7)	Fisher’s exact test	.223
Delayed wound healing, n (%)	6 (10.7)	4 (3.6)	Fisher’s exact test	.085
Cardiovascular events, n (%)	3 (5.4)	4 (3.6)	Fisher’s exact test	.687
Delirium, n (%)	9 (16.1)	7 (6.3)	Fisher’s exact test	.052
Readmission, n (%)	4 (7.1)	3 (2.7)	Fisher’s exact test	.223
Reoperation, n (%)	2 (3.6)	1 (0.9)	Fisher’s exact test	.258

### 3.5. Comparison of secondary outcomes between the 2 groups

Patients in the malnutrition group had a significantly longer length of hospital stay than those in the non-malnutrition group (12.8 ± 4.7 vs 10.9 ± 3.9 days, t = 2.61, *P* = .011). The rate of perioperative blood transfusion was also significantly higher in the malnutrition group (32.1% vs 17.9%, χ^2^ = 4.35, *P* = .037). In contrast, although ICU admission occurred more frequently in patients with malnutrition than in those without malnutrition (14.3% vs 6.3%), the difference did not reach statistical significance (χ^2^ = 2.02, *P* = .155; Table 4).

**Table 4:** Comparison of secondary in-hospital outcomes between the malnutrition group and the matched non-malnutrition group.

Variable	Malnutrition group (n = 56)	Non-malnutrition group (n = 112)	Test statistic	*P* value
Length of hospital stay, d	12.8 ± 4.7	10.9 ± 3.9	*t* = 2.61	.011
ICU admission, n (%)	8 (14.3)	7 (6.3)	χ^2^ = 2.02	.155
Perioperative blood transfusion, n (%)	18 (32.1)	20 (17.9)	χ^2^ = 4.35	.037

ICU = intensive care unit.

### 3.6. Univariable logistic regression analysis

Univariable logistic regression showed that older age, lower BMI, coronary artery disease, chronic obstructive pulmonary disease, ASA class III–IV, greater intraoperative blood loss, perioperative blood transfusion, lower hemoglobin, lower albumin, lower total lymphocyte count, lower total protein, higher C-reactive protein, and GLIM-defined malnutrition were significantly associated with postoperative complications (all *P* < .05). In contrast, sex, smoking history, alcohol consumption history, hypertension, diabetes mellitus, chronic kidney disease, cerebrovascular disease, other major comorbidities, surgical procedure, anesthesia modality, operative time, and serum creatinine were not significantly associated with postoperative complications (all *P* > .05; Table 5).

**Table 5:** Univariable logistic regression analysis of factors associated with postoperative complications after hip arthroplasty.

Variable	OR	95% CI	*P* value
Age, per 1-yr increase	1.06	1.01–1.11	.010
Male sex	1.05	0.52–2.11	.894
BMI, per 1 kg/m^2^ increase	0.81	0.71–0.93	.003
Smoking history	1.44	0.70–2.95	.328
Alcohol consumption history	1.04	0.46–2.34	.930
Hypertension	1.71	0.86–3.40	.124
Diabetes mellitus	1.79	0.85–3.77	.126
Coronary artery disease	2.28	1.03–5.06	.043
Chronic obstructive pulmonary disease	2.62	1.01–6.77	.048
Chronic kidney disease	2.27	0.84–6.14	.106
Cerebrovascular disease	1.84	0.82–4.12	.138
Other major comorbidities	1.75	0.78–3.94	.176
ASA class III–IV	2.93	1.42–6.03	.004
THA (vs HA)	0.62	0.30–1.28	.181
General anesthesia	1.61	0.68–3.83	.281
Operative time, per 1-min increase	1.02	1.00–1.04	.094
Intraoperative blood loss, per 10 mL increase	1.04	1.01–1.08	.015
Perioperative blood transfusion	3.14	1.50–6.58	.002
Hemoglobin, per 1 g/L increase	0.96	0.93–0.99	.002
Albumin, per 1 g/L increase	0.81	0.74–0.90	<.001
Total lymphocyte count, per 1 × 10^9^/L increase	0.31	0.14–0.69	.004
Total protein, per 1 g/L increase	0.91	0.86–0.97	.003
Serum creatinine, per 1 μmol/L increase	1.01	0.99–1.02	.313
C-reactive protein, per 1 mg/L increase	1.06	1.01–1.11	.010
GLIM-defined malnutrition	2.92	1.47–5.79	.002

ASA = American Society of Anesthesiologists, BMI = body mass index, CI = confidence interval, GLIM = global leadership initiative on malnutrition, HA = hemiarthroplasty, OR = odds ratio, THA = total hip arthroplasty.

### 3.7. Multivariable logistic regression analysis

Variables with statistical significance in the univariable analysis, together with clinically relevant covariates, were entered into the multivariable logistic regression model. After adjustment, GLIM-defined malnutrition remained independently associated with postoperative complications after hip arthroplasty (adjusted odds ratuo [OR] = 2.74, 95% confidence interval [CI]: 1.23–6.09, *P* = .013). In addition, ASA class III–IV (adjusted OR = 2.21, 95% CI: 1.01–4.86, *P* = .048), greater intraoperative blood loss (adjusted OR = 1.03 per 10 mL increase, 95% CI: 1.00–1.06, *P* = .041), perioperative blood transfusion (adjusted OR = 2.63, 95% CI: 1.16–5.96, *P* = .021), lower albumin (adjusted OR = 0.86, 95% CI: 0.76–0.97, *P* = .015), and higher C-reactive protein (adjusted OR = 1.04, 95% CI: 1.00–1.09, *P* = .049) were also independently associated with postoperative complications. Other variables, including age, BMI, coronary artery disease, chronic obstructive pulmonary disease, hemoglobin, total lymphocyte count, and total protein, were not statistically significant in the adjusted model (Table 6).

**Table 6:** Multivariable logistic regression analysis of factors associated with postoperative complications after hip arthroplasty.

Variable	Adjusted OR	95% CI	*P* value
Age, per 1-yr increase	1.04	0.99–1.10	.118
BMI, per 1 kg/m^2^ increase	0.89	0.76–1.03	.124
Coronary artery disease	1.72	0.71–4.18	.230
Chronic obstructive pulmonary disease	1.94	0.66–5.68	.226
ASA class III–IV	2.21	1.01–4.86	.048
Intraoperative blood loss, per 10 mL increase	1.03	1.00–1.06	.041
Perioperative blood transfusion	2.63	1.16–5.96	.021
Hemoglobin, per 1 g/L increase	0.98	0.95–1.01	.136
Albumin, per 1 g/L increase	0.86	0.76–0.97	.015
Total lymphocyte count, per 1 × 10^9^/L increase	0.52	0.19–1.39	.190
Total protein, per 1 g/L increase	0.96	0.89–1.03	.266
C-reactive protein, per 1 mg/L increase	1.04	1.00–1.09	.049
GLIM-defined malnutrition	2.74	1.23–6.09	.013

ASA = American Society of Anesthesiologists, BMI = body mass index, CI = confidence interval, GLIM = global leadership initiative on malnutrition, OR = odds ratio.

## 4. Discussion

The present study evaluated the association between GLIM-defined malnutrition and short-term postoperative outcomes in older patients undergoing hip arthroplasty. The principal findings were as follows. First, after 1:2 matching for age, sex, surgical year, and primary indication for hip arthroplasty, patients with malnutrition still showed a less favorable nutritional and inflammatory profile, characterized by lower body weight, lower BMI, lower hemoglobin, albumin, total lymphocyte count, and total protein, together with higher CRP levels. Second, the malnutrition group had a significantly higher incidence of overall postoperative complications, a longer hospital stay, and a higher perioperative transfusion rate. Third, multivariable logistic regression demonstrated that GLIM-defined malnutrition remained independently associated with postoperative complications after adjustment for other relevant perioperative factors. These findings indicate that GLIM-based nutritional assessment captures clinically meaningful vulnerability that is not fully explained by age, surgical indication, or selected comorbid conditions.

The observed association between malnutrition and adverse postoperative outcomes is consistent with recent literature in both arthroplasty and hip-fracture populations. In a 2023 study of elderly patients undergoing elective THA, Fernández Miró and colleagues reported that GLIM-defined malnutrition was associated with postoperative complications and longer length of stay, and that malnourished patients had lower BMI and lower albumin concentrations. A recent 2025 cohort study of older adults undergoing hip arthroplasty also suggested that preoperative nutritional disorders, particularly undernutrition and sarcopenia-related phenotypes, were linked to less favorable short-term recovery. In parallel, contemporary reviews of older hip-fracture populations have emphasized that malnutrition is highly prevalent and is associated with delirium, pressure injury, prolonged hospitalization, and loss of independence. Taken together, the present results extend this pattern of evidence to a matched cohort of older patients undergoing hip arthroplasty for mixed indications, supporting the external validity of the current findings.^[11,12]^ Several mechanisms may explain why GLIM-defined malnutrition was associated with postoperative complications in this setting. The GLIM framework combines phenotypic abnormalities, such as low BMI and weight loss, with etiologic features including reduced intake and inflammatory burden. This approach is clinically relevant because postoperative recovery after hip arthroplasty requires adequate metabolic reserve, preserved muscle function, and sufficient protein availability to support immune defense, tissue repair, and mobilization. Patients with malnutrition are more likely to enter the perioperative period with depleted lean mass, impaired functional reserve, and reduced tolerance of surgical stress. In older adults, these deficits may be amplified by anabolic resistance, multimorbidity, and reduced physiologic resilience. Current ESPEN guidance also emphasizes that malnutrition and underfeeding increase the risk of postoperative complications and supports early identification of patients at nutritional risk, integration of nutrition into perioperative care, and early oral or artificial nutritional support when appropriate.^[13]^

The pattern of individual complications in the present study is also clinically informative. Although the study was not powered to detect significant differences for every specific event, delirium was the most frequent complication in both groups and occurred more often in the malnutrition group, with borderline statistical significance. This trend is biologically plausible. Delirium after hip surgery in older adults is multifactorial, but inflammatory activation, anemia, hypoalbuminemia, impaired cerebral reserve, and perioperative stress are recognized contributors. A 2023 retrospective cohort study found that an elevated preoperative CRP-to-albumin ratio was independently associated with postoperative delirium and mortality in elderly patients undergoing hip-fracture surgery. Likewise, recent literature on hip-fracture recovery has continued to identify malnutrition and inflammation as factors associated with delirium and other adverse geriatric outcomes. The higher, although nonsignificant, rates of pneumonia and delayed wound healing observed in our malnutrition group are similarly consistent with impaired host defense, diminished respiratory muscle performance, and delayed collagen synthesis in nutritionally compromised patients.^[14,15]^

The secondary outcome findings further support the clinical relevance of preoperative malnutrition. Patients with malnutrition had a longer hospital stay and required perioperative transfusion more often. These findings are consistent with the view that malnutrition is not merely a biochemical abnormality but a marker of reduced resilience across the perioperative trajectory. Longer hospitalization may reflect delayed mobilization, slower wound recovery, a greater need for medical stabilization, or more frequent monitoring for complications. The higher transfusion rate may be partly explained by the lower baseline hemoglobin observed in the malnutrition group, but it may also reflect poorer tolerance of blood loss and less physiologic reserve. Recent arthroplasty research has reported that undernutrition and sarcopenia-related phenotypes are associated with larger postoperative hemoglobin decline and slower early functional recovery, which is concordant with the present observation that nutritionally vulnerable patients consumed greater perioperative resources.^[16,17]^ The multivariable model identified GLIM-defined malnutrition as an independent factor associated with postoperative complications, together with ASA class III–IV, greater intraoperative blood loss, perioperative transfusion, lower albumin, and higher CRP. This combination of predictors is clinically coherent. ASA status reflects overall systemic disease burden and perioperative vulnerability. Blood loss and transfusion may indicate surgical complexity and may also contribute to physiologic stress, hemodynamic instability, and inflammatory activation. Albumin and CRP represent complementary aspects of nutritional reserve and systemic inflammation. Recent studies in hip-fracture and arthroplasty populations have likewise highlighted the prognostic role of albumin-related and inflammation-related markers. For example, preoperative CRP/albumin ratio has been associated with postoperative delirium and mortality after hip-fracture surgery, while recent arthroplasty and HA studies have linked albumin-related nutritional deficits to wound complications, prolonged recovery, and worse short-term outcomes. Accordingly, the current findings suggest that GLIM-defined malnutrition should be viewed not in isolation, but as part of a broader perioperative risk phenotype that integrates nutrition, inflammation, and operative stress.^[18,19]^

Another relevant observation is that the malnutrition group contained a higher proportion of patients living alone. Although this variable was not retained as an independent factor in the final model, it may still be clinically meaningful. Living alone may serve as a proxy for reduced social support, less assistance with food procurement and meal preparation, or delayed recognition of nutritional decline before admission. In older surgical patients, nutritional vulnerability often develops within a broader context of frailty, functional limitation, and social disadvantage. Recent guideline updates increasingly encourage perioperative assessment that extends beyond isolated laboratory markers to include frailty, sarcopenia, and broader functional context. From this perspective, the present data support a more comprehensive preoperative screening strategy in older hip arthroplasty candidates. These findings have several practical implications. First, routine preoperative nutritional assessment using a structured framework such as GLIM may improve risk stratification in older patients scheduled for hip arthroplasty. Second, patients identified as malnourished may benefit from targeted perioperative optimization, including dietitian referral, protein-energy supplementation, correction of reversible anemia, and closer surveillance for delirium, respiratory complications, skin breakdown, and delayed wound healing. Third, the co-occurrence of low albumin and elevated CRP in the present study suggests that combined nutrition-inflammation assessment may be particularly useful when prioritizing patients for intensified perioperative support. Recent guidance and observational evidence support early nutritional intervention as part of perioperative care pathways, especially in older adults at nutritional risk.^[20,21]^

Several limitations should be acknowledged. First, this was a retrospective single-center study, and residual confounding cannot be excluded despite matching and multivariable adjustment. Specifically, selection bias may have been introduced because only patients with complete clinical and nutritional data were included; those with incomplete records − often frailer or more acutely ill older adults − may have been systematically excluded, potentially altering the observed association. Information bias is another concern, as the GLIM diagnostic components (particularly reduced food intake and muscle mass assessment) relied on retrospective chart review rather than standardized prospective measurements, which might lead to misclassification of nutritional status. Moreover, important unmeasured confounders − such as preoperative functional status, frailty scores, and cognitive function − were not available for analysis, and their absence may have inflated the independent effect of GLIM-defined malnutrition. The sample size was moderate, and the malnutrition group comprised only 56 patients after matching. A formal a priori power calculation was not performed because of the retrospective design; however, a post hoc power analysis based on the observed complication rates (42.9% vs 20.5%) yielded 82% power at α = 0.05 to detect the difference in overall complications, but the study was clearly underpowered for analyses of individual complications. Therefore, the nonsignificant between-group differences for specific events (e.g., pneumonia, delayed wound healing) should be interpreted with caution and may reflect type II error.

## 5. Conclusion

In conclusion, this study indicates that GLIM-defined malnutrition is independently associated with postoperative complications in older patients undergoing hip arthroplasty. Malnourished patients also exhibited a longer hospital stay, a higher transfusion rate, and a less favorable preoperative nutritional-inflammatory profile. These findings support incorporation of GLIM-based nutritional screening into routine perioperative evaluation for older hip arthroplasty patients and provide a rationale for prospective studies testing whether structured nutritional optimization can reduce postoperative morbidity in this population.

## Author contributions

**Conceptualization:** Zhixing Lu, Wenwei Xie.

**Data curation:** Zhixing Lu, Wenwei Xie.

**Formal analysis:** Zhixing Lu, Wenwei Xie.

**Funding acquisition:** Wenwei Xie.

**Investigation:** Wenwei Xie.

**Writing − original draft:** Wenwei Xie.

**Writing − review & editing:** Wenwei Xie.
